# Preparation and characterization of templated porous carbons from sucrose by one-pot method and application as a CO_2_ adsorbent

**DOI:** 10.3906/kim-2012-11

**Published:** 2021-08-27

**Authors:** Meltem GÜRBÜZ, Fatma TÜMSEK

**Affiliations:** 1 Department of Chemical Engineering, Faculty of Engineering and Architecture, Eskişehir Osmangazi University, Eskişehir Turkey

**Keywords:** Porous carbon, templated synthesis, one-pot method, CO_2_adsorption

## Abstract

The templated porous carbons were prepared from sucrose by one-pot method. In this method in which the pre-synthesis of the hard template is eliminated, the porous carbons were produced by organic-inorganic self-assembly of sucrose, tetraethyl ortosilicate (TEOS), Pluronic P123 and n-butanol in an acidic medium, and subsequent carbonization. The synthesis parameters such as sucrose amount, TEOS molar ratio and carbonization temperature were evaluated for describing their effects on the pore structures of the synthesized carbons. The prepared porous carbons were characterized by N_2_ adsorption, thermogravimetric analysis (TGA), Raman spectroscopy, X-ray diffraction (XRD), field emission scanning electron microscopy (FESEM), and transmission electron microscopy (TEM) techniques. The carbon dioxide adsorption uptakes of the obtained porous carbons were determined at 1 bar and 273 K. The templated carbon obtained with the lowest TEOS molar ratio exhibited the highest BET surface area of 1289 m^2^/g and micropore volume of 0.467 cm^3^/g, and showed the highest CO_2_ uptake of 2.28 mmol/g.

## 1. Introduction

Nowadays, climate change due to the increase in greenhouse gas emissions accumulated in the atmosphere is a major threat to the world. Carbon dioxide (CO_2_) is one of the gases that has a major share in global warming. The share of CO_2_ in the greenhouse effect factors is about 55% [1–2]. CO_2_ emissions, as a result of from industry, power plants and combustion of fossil fuels, which are worldwide problems [3]. Therefore, reducing the CO_2_ concentration in the atmosphere has recently been an important issue. For that reason, the development of CO_2_ capture and storage techniques is extremely important [4–5].

Recently, solid adsorption, liquid absorption and membrane separation can be mentioned as the main methods for CO_2_ capture [2,6]. Among these methods, CO_2_ adsorption is a prominent method due to its advantages such as low energy requirement, low cost, ease of application, and regeneration of the adsorbent [7–10]. In order for CO_2_ adsorption to be efficient, it is important to develop an adsorbent with a high adsorption capacity, selectivity, kinetics and stability [7,11]. Commonly used solid adsorbents for CO_2_ capture are zeolites [12–13], silica [14], clay minerals [15], metal-organic frameworks [16–19], porous polymer materials [20–22], and carbon materials [23–29].

Because the carbon materials have properties such as large surface areas and pore volumes, hydrophobicity of the surface, relatively easy regeneration, good thermal and mechanical stability, and inexpensive preparation costs, they stand out for CO_2_ adsorption among other adsorbents [1–5,30]. Activated carbons are the most widely used carbon materials in this area [30–35]. However, since activated carbons are produced from various raw materials and by various methods, they offer an uncontrollable pore structure and surface properties. The carbon adsorbents with controlled porous structure were prepared by using different synthesis ways like template method. The hard or soft template carbonization methods have attracted much attention to prepare porous carbon materials with desirable properties [4,36]. In particular, the ordered porous carbons which have quite narrow and uniform pore size distribution can be prepared with this method and they are considered to be very suitable CO_2_ adsorbents [37].

The template synthesis of ordered mesoporous carbons was first reported by Ryoo and coworkers [38,39] using mesoporous silica materials such as MCM-48 and SBA-15 as hard templates and sucrose as the carbon source. Although the ordered mesoporous carbons obtained by this method have large surface areas, large pore volumes, and narrow pore size distributions, the process of preparing these carbons by the hard template method is time-consuming and includes multiple steps such as the need to prepare the hard template first and then impregnation. Later, a one-pot method that eliminates the pre-synthesis step of silica and allows the simultaneous formation of carbon and silica was presented. Ting and co-workers [40] reported a simple one-pot method for preparing of hexagonally ordered mesoporous carbon. In their method, a composite was formed in a one-pot using sucrose, tetraethyl ortosilicate (TEOS), and Pluronic P123 via organic-inorganic self-assembly under acidic conditions, and then mesoporous carbons were obtained by removing silica following carbonization. They showed that the structural properties of ordered mesoporous carbons can be adjusted simply by changing the reaction parameters and compositions [40]. Prabhu et al. [41] presented a work that involves the simultaneous production of KIT-6 silica and carbon by one-pot method. In this method, P123/n-butanol/sucrose/silica composite directly catalyzed by H_2_SO_4_ in the synthesis mixture was generated, and the mesoporous carbon was prepared after carbonization followed by silica removal. Thus, the mesoporous carbon with three-dimensional cubic symmetry was obtained [41]. In their report, the synthesis of this carbon was carried out under a specific condition only; some important points such as the effects of the synthesis parameters and of the amounts of components in the synthesis mixture on the structure of the resulting carbons were not addressed. In addition, it was emphasized that the synthesis conditions and compositions still need to be optimized. However, to the best of our knowledge, no reports have been found in the published literature regarding the effects of synthesis conditions of mesoporous carbon with three-dimensional cubic symmetry produced by the one-pot method. To overcome this gap, the synthesis parameters and compositions were investigated in this study by employing one-pot technique for these carbons development. To ensure the widespread use of porous carbons, it is important to develop simple synthesis methods that directly lead to their formation and to optimize these synthesis methods. 

In particular, the studies based on high performance adsorbents for carbon dioxide retention have attracted great attention. Currently, the researches focus on using low-cost resources to develop efficient and economical synthesis pathways for ideal performance sorbents. The present study is thought to be the first attempt to introduce CO_2_ adsorption potentials of the templated porous carbons which produced using one-pot method a simplified technique, from sucrose as an economical and abundant source.

In this study, templated carbons were prepared using sucrose as a carbon precursor and tetraethyl orthosilicate (TEOS) as silica precursor by one-pot method. In this method, the organic-inorganic self-assembly of sucrose, TEOS, Pluronic P123, and n-butanol in acidic medium was performed, thus the pre-synthesis of hard template was eliminated. In addition, the porous carbons produced from sucrose can be advantageous since sucrose is a low-cost, environmentally friendly, and abundantly available chemical. The CO_2_ adsorption potentials of the templated porous carbons were determined. The effects of synthesis parameters such as carbonization temperature, molar ratio of TEOS and amount of sucrose on the properties of carbons were also investigated. This examination was supported by different characterization tests including field emission scanning electron microscopic (FESEM) imaging, transmission electron microscopic (TEM) imaging, thermogravimetric analysis (TGA), Raman spectroscopy, X-ray diffraction (XRD), surface area, and porosity analysis.

## 2. Materials and methods

### 2.1. Chemicals

In the synthesis of carbons, sucrose (Carlo Erba), tetraethyl orthosilicate (TEOS) (Acros Organics), triblock copolymer (Pluronic P123 MW = 5800, Sigma Aldrich), n-butanol (VWR Chemicals), and sulfuric acid (H_2_SO_4_) (Fluka) were used as the carbon source, silica source, structure directing agent, cosolvent, and catalyst, respectively. Hydrochloric acid (HCl) (Merck) was used to provide the acidic medium and hydrofluoric acid (HF) (Fisher Chemical) was used to remove silica. All of the chemicals used in the study were analytical grade and they were used as received.

### 2.2. Synthesis of templated porous carbons

The templated porous carbons were synthesized via the one-pot method with some modifications according to the method found in the related literature [41]. In a typical synthesis, 4 g of P123 was added to the acidic solution containing 7.9 g of HCl and 144 g of water and the mixture was stirred at room temperature for 3 h. Then, 4 g of n-butanol was added to the same solution and stirred for a further 2 h. To the resulting homogeneous solution was added 1 g of H_2_SO_4_ and a certain amount of sucrose (0.6, 0.73, and 0.85 g) and again stirred for 2 h. A certain molar ratio (0.52, 1.70, and 2.88) of TEOS was added to this solution and the mixture was allowed to stand at 35 °C for 24 h under vigorous stirring. This solution was aged hydrothermally at 100 °C for 24 h in polypropylene bottles. Subsequently, it was kept in the oven at 100 °C for 6 h and then at 160 °C for 6 h. The resulting composite powder was subjected to carbonization under nitrogen flow at various temperatures (766, 850, and 934 °C) for 3 h. A 10% HF solution was used to remove the silica from the resulting solid and the carbonized solid was allowed to stand in this solution for 24 h. It was then filtered, washed with ethanol, and dried at 100 °C. Since it was aimed to investigate the effects of sucrose content, TEOS molar ratio and carbonization temperature on the properties of the formed carbons, these conditions were changed at the values indicated in brackets and synthesis was applied. The porous carbons are named as S-x/T-y/C-z, where x is the amount of sucrose and y is the molar ratio of TEOS and z is the carbonization temperature. All notations of the prepared carbon adsorbents along with their synthesis conditions are given in Table 1.

**Table 1:** Carbon samples and their synthesis conditions.

Carbon	Sucrose amount (g)	TEOS molarratio	Carbonization temperature (°C)
S-0.60/T-1.70/C-850	0.60	1.70	850
S-0.73/T-1.70/C-850	0.73	1.70	850
S-0.85/T-1.70/C-850	0.85	1.70	850
S-0.73/T-0.52/C-850	0.73	0.52	850
S-0.73/T-2.88/C-850	0.73	2.88	850
S-0.73/T-1.70/C-766	0.73	1.70	766
S-0.73/T-1.70/C-934	0.73	1.70	934

### 2.3. Characterization

The pore volumes and specific surface areas of the porous carbons were determined by analyzing the nitrogen adsorption-desorption isotherms which found at 77 K using an Autosorb 1-C (Quantachrome, USA) analyzer. Before the analysis, the carbon samples were degassed at 300 °C for 3 h under vacuum. The Brunauer–Emmett–Teller (BET) method was used to determine the specific surface areas of carbons and Barret-Joyner-Halenda (BJH) method was applied to adsorption isotherm to determine the pore size distributions of carbons. The total pore volume (V_total_) was obtained from the adsorbed volume of N_2_ at relative pressure of 0.99. The micropore volumes (V_micro_) were determined by using the Dubinin–Radushkevich (DR) method. The X-ray diffraction (XRD) patterns were collected by Panalytical Empyrean at a scanning speed of 5 °/min and a scanning angle of 10–70 ° for analyzing the structures of carbons. In addition, Raman spectroscopy (Renishaw inVia) was also used with a laser wavelength of 532 nm and in a scanning range of 100–4000 cm^–1^. Field emission scanning electron microscopy (FESEM) (Hitachi Regulus 8230) and transmission electron microscopy (TEM) (Hitachi HT7800) were used to characterize the surface morphology and microstructure of carbons. Elemental composition was demonstrated by energy dispersive X-ray spectroscopy (EDS). Thermal behavior of carbon was performed on a thermal analyzer (Perkin Elmer STA 8000) at 10 °C/min. The CO_2_ adsorption isotherms of the porous carbons were determined at a temperature of 273 K and in a pressure range of 0–1 bar by using a volumetric adsorption apparatus (Quantachrome Autosorb 1-C).

## 3. Results and discussion 

The characterization results obtained for carbons produced under the conditions specified in Table 1 and the results for CO_2_ adsorption are presented in the following sections.

### 3.1. Characterization of porous carbons

**Figure 1:**
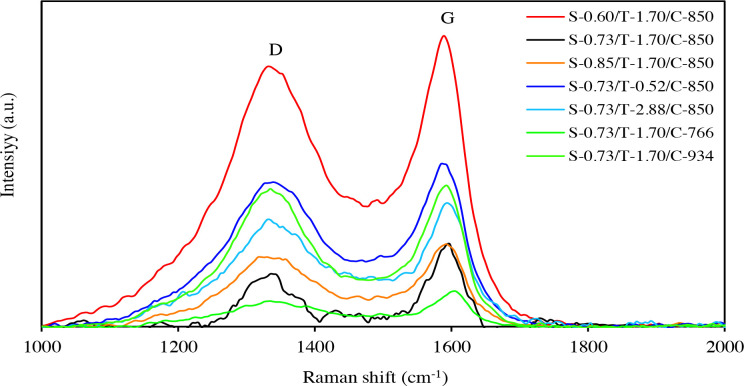
Raman spectra for porous carbons.

The molecular morphology characterization of carbon materials was performed by Raman spectroscopy. The Raman spectra for all carbon samples are given in Figure 1. The Raman analysis of the carbon samples showed two main peaks at around 1335 and 1590 cm^–1^. The G band at 1590 cm^–1^ indicates that the carbon samples contain sp^2^ bonded carbon in planar sheets. This represents the presence of ordered carbon structure. The broad band at 1335 cm^–1^ is known as the D band and this is due to a hybridized vibrational mode associated with graphene edges in disordered carbon with structural defects and disorders [8,26,42–43]. The intensity ratio of D to G band, I_D_/I_G_, is usually used to characterize the graphitization degree of carbon materials. The I_D_/I_G_ ratios for the carbon samples are given in Table 2. A higher I_D_/I_G_ ratio indicates a lower degree of graphitization. The results show that the S-0.73/T-1.70/C-850 carbon has the highest and S-0.73/T-1.70/C-934 carbon has the lowest graphitic degree. For all porous carbon samples, the I_D_/I_G_ ratios are close to 1 and found to be well compatible with similar mesoporous carbon materials [44–46].

**Table 2:** Structural parameters of porous carbons.

Carbon	ID/IG	d002 (nm)
S-0.60/T-1.70/C-850	0.89	0.3543
S-0.73/T-1.70/C-850	0.64	0.3600
S-0.85/T-1.70/C-850	0.83	0.3687
S-0.73/T-0.52/C-850	0.88	0.3628
S-0.73/T-2.88/C-850	0.87	0.3737
S-0.73/T-1.70/C-766	0.72	0.3535
S-0.73/T-1.70/C-934	0.98	0.3657

**Figure 2:**
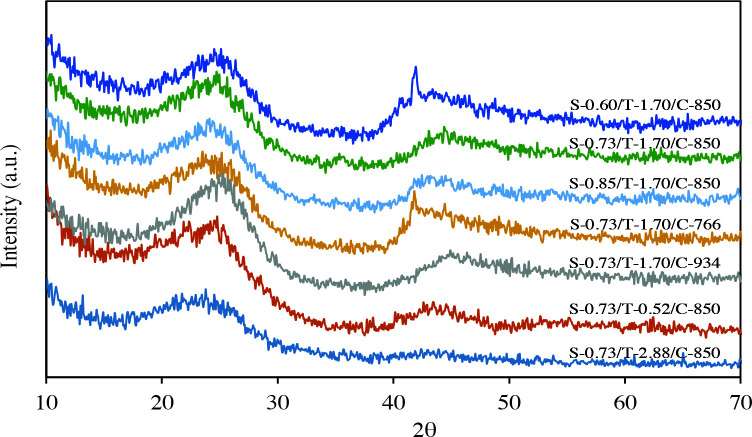
XRD patterns of porous carbons.

The XRD patterns of templated porous carbons are presented in Figure 2. Two broad peaks at around 25 ° and around 44 ° shown in the XRD patterns can be attributed to (0 0 2) and (1 0 0) plane for all porous carbons [8,42–43]. These peaks are typical for amorphous carbons [23]. The (0 0 2) peak for carbons indicates inter-planar stacking structures of graphitic carbon [42]. The (1 0 0) diffraction peak is caused by the disordered structure of carbons [1]. The values of d-spacing (d_002_) from XRD measurement are given in Table 2. The d_002_ of porous carbons increased from 0.3535 nm to 0.3657 nm as the carbonization temperature increases. A similar situation is observed with an increase in the amount of sucrose. Again, the increase of the TEOS molar ratio to 2.88 also caused an increase in the d-spacing.

**Figure 3:**
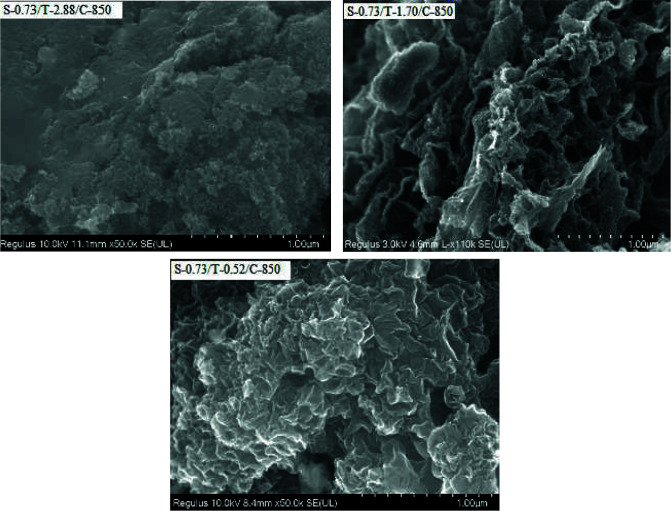
FESEM images of the porous carbons.

**Figure 4:**
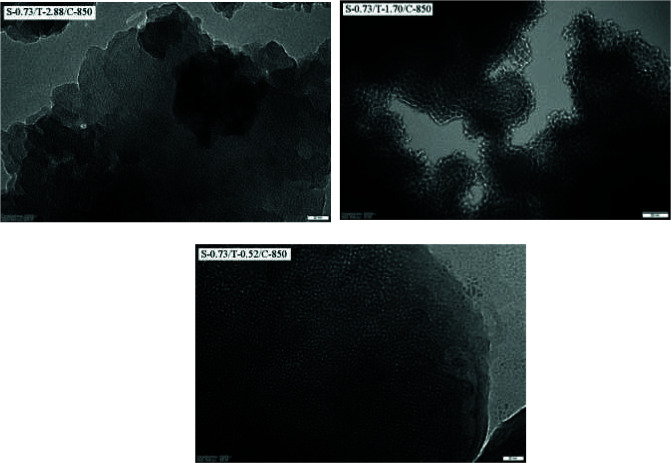
TEM images of the porous carbons.

**Figure 5:**
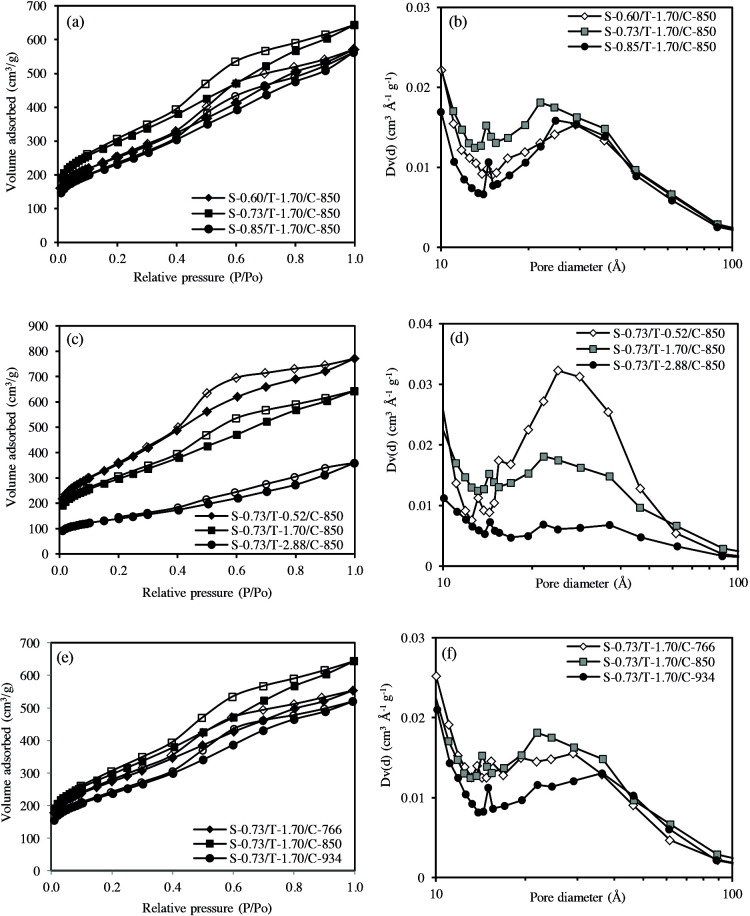
The adsorption-desorption isotherms (a, c, e) and pore size distributions (b, d, f) of porous carbons.

Figure 3 shows the FESEM images of the carbon samples prepared at different TEOS molar ratio. The pores and folds are observed on the carbon surfaces. The FESEM image of S-0.73/T-2.88/C-850 carbon prepared with the highest TEOS molar ratio shows a smoother surface with less porous structure. The surface appears to become rougher as the TEOS molar ratio decreases. The porous characteristics of the carbons can be viewed directly in the TEM images in Figure 4. In this figure, the mesopores of 2–4 nm in diameter can be distinguished on the carbon surfaces. The TEM image of the S-0.73/T-1.70/C-850 carbon sample also shows cubic arrays of structure. There are relatively regular distributed pores which is a typical characteristic of ordered porous carbons. Some disordered pores are also shown at edge positions. S-0.73/T-0.52/C-850 carbon exhibits a structure with more uniform pore size compared to other samples. This result is in good agreement with pore size distribution described later in this article (Figures 5a-d). The EDS analysis determined with FESEM indicated that the carbon content of the samples is at about 94 wt%, O content is at about 5 wt% and Si content is at about 0.23 wt%. This result proves that the silica is almost completely removed from the structure.

The nitrogen adsorption-desorption isotherms of porous carbons and pore size distributions are shown in Figure 5. The specific surface areas and pore properties of the porous carbons are given in Table 3. The adsorption-desorption isotherms of all carbons can be ascribed to type IV according to the IUPAC classification. This type of the isotherm is typical for materials containing mesopores, besides micropores. This is also seen in pore size distributions. All porous carbons show well developed pore size distribution below 10 nm, indicating the presence of both micropores and mesopores. Almost all isotherms show type H2 hysteresis loops. Such loops are characterized by a long and nearly flat adsorption branch and a steep desorption branch. It is said that the pore structures of materials showing such isotherms are complex and interconnected. As the molar ratio of TEOS increased, the amount of nitrogen adsorbed decreased and the shape of the hysteresis loop was similar to the H4 type (Figure 5c). It can be concluded that the molar ratio of TEOS changes the pore properties of the formed carbons. This result can be seen from Table 3. When the TEOS molar ratio increased from 0.52 to 2.88, the specific surface areas and pore volumes sharply decreased from 1289 to 495 m^2^/g and from 1.196 to 0.557 cm^3^/g, respectively. The effects of sucrose amount and carbonization temperature on the pore properties of carbons were not as significant as the molar ratio of TEOS.

**Table 3:** Specific surface areas and pore properties of porous carbons and CO2 uptakes.

Carbon	SBET (m2/g)	Vtotal (cm3/g)	Vmicro (cm3/g)	Vmeso(cm3/g)	Dp (nm)	CO2 uptake at 273 K,1 bar (mmol/g)
S-0.60/T-1.70/C-850	900	0.886	0.340	0.546	3.98	1.85
S-0.73/T-1.70/C-850	1052	0.998	0.398	0.600	3.80	1.87
S-0.85/T-1.70/C-850	827	0.873	0.310	0.563	4.24	1.77
S-0.73/T-0.52/C-850	1289	1.196	0.467	0.729	3.71	2.28
S-0.73/T-2.88/C-850	495	0.557	0.191	0.366	4.57	1.27
S-0.73/T-1.70/C-766	977	0.858	0.372	0.486	3.56	1.95
S-0.73/T-1.70/C-934	845	0.806	0.324	0.482	3.87	1.63

**Figure 6:**
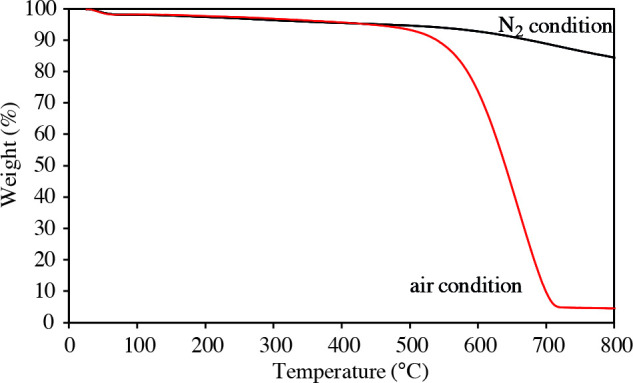
Thermogravimetric analysis for the S-0.73/T-1.70/C-850 carbon sample.

The thermal stability of the obtained porous carbons was examined by TGA analysis. Thermal behaviors of the S-0.73/T-1.70/C-850 carbon under N_2_ and air atmosphere are shown in Figure 6. The curve obtained under N_2_ flow exhibits three weight loss steps. The initial step weight loss is about 1.8% at below 100 °C, which is mainly attributed to the loss of moisture outside the surface and adsorbed water in the structure. The second step shows the weight loss of 3.7% at about 500 °C. The largest weight loss of 10.45% is in the third stage from 500 to 800 °C. As compared with that in N_2_ atmosphere, the template porous carbon under air flow shows a significant weight loss which represents the oxidation of carbon in the temperature range of 500–700 °C. The mass content in TGA curve of carbon under air atmosphere is close to zero at temperature above 700 °C. The weight of residue was only 4.5% indicating that most components were burnt in air. In addition, little residue showed that the dissolution of silica was nearly complete. The results confirmed that the template porous carbon materials have high thermal stability in N_2_ and air.

### 3.2. CO2 adsorption 

**Figure 7:**
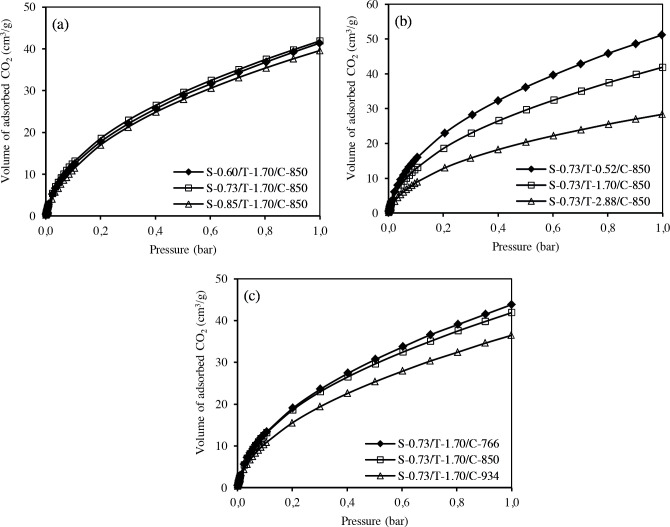
The CO2 adsorption isotherms of templated porous carbons at 273 K.

Figure 7 shows CO_2_ adsorption isotherms of porous carbons at 273 K. The corresponding uptakes at 1 bar are presented in Table 3. The effects of sucrose amount on the CO_2_ adsorption is shown in Figure 7a. The CO_2_ adsorption isotherms for carbons prepared at different amount of sucrose are very close to each other. The effects of TEOS molar ratio are displayed in Figure 7b. Increasing of the TEOS molar ratio from 0.52 to 2.88 gives rise to negative impacts on CO_2_ uptake. In Figure 7c, carbon samples carbonized at 766 and 850 °C have very close CO_2_ adsorption capacities, however CO_2_ adsorption amount decreases when the carbonization temperature reaches to 934 °C. 

**Figure 8:**
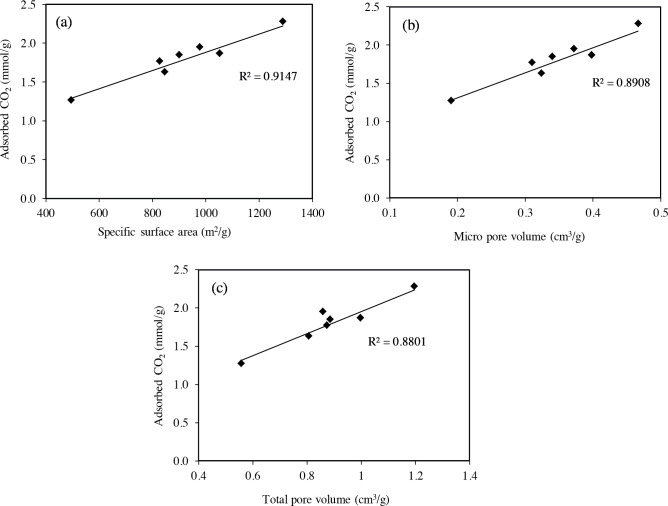
The relationship of adsorbed amount of CO2 with (a) specific surface area, (b) micropore volume and (c) total pore volume of the templated carbons.

Figure 8 shows the relationship of the adsorbed amount of CO_2_ at 1 bar with the pore properties of the templated porous carbons. As can be seen in Figure 8, there is a nearly linear relationship between the CO_2_ uptakes and pore properties. A very similar trend is observed for the adsorbed CO_2_ amount in relation to the specific surface area and the micropore volume (Figures 8a and b). These pore properties have more linear relationship with CO_2_ uptake as compared with total pore volume (Figure 8c). As the micropore volume and surface area presented by porous carbons increased, the CO_2_ adsorption amount increased. 

**Figure 9:**
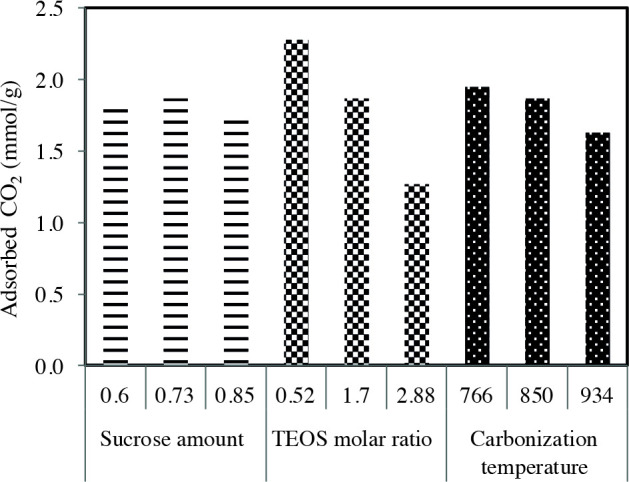
The change of CO_2_ uptake with synthesis parameters.

The effect of the synthesis parameters on the CO_2_ adsorption capacities of the templated carbons is presented in Figure 9. The results show that the TEOS molar ratio has more influence on the CO_2_ adsorption amount. While the S-0.73/T-0.52/C-850 carbon prepared with the lowest TEOS molar ratio exhibits the highest capacity of 2.28 mmol/g, the S-0.73/T-2.88/C-850 carbon synthesized with the highest TEOS molar ratio has the lowest CO_2_ uptake of 1.27 mmol/g. The higher CO_2_ adsorption capacity of S-0.73/T-0.52/C-850 carbon can be attributed to the higher surface area and micropore volume of this carbon compared to other samples. As mentioned earlier, the CO_2_ capacity is directly related to the micropore volumes and surface areas of the carbons. Since TEOS is used as a source of silica in the synthesis of these carbons, it is thought that increasing the amount of TEOS leads to an increase in the pore wall thickness of the silica formed simultaneously with the carbon structure. It was concluded that when the TEOS molar ratio is high, the porous carbon formed after the silica was removed from the structure had larger pores and therefore the surface area of carbon decreased. When the TEOS molar ratio was 2.88, the average pore size of the formed carbon S-0.73/T-2.88/C-850 had the largest value of 4.57 nm among the synthesized carbons and the surface area had the lowest value of 495 m^2^/g. Increasing the amount of sucrose did not have a significant effect on the CO_2_ adsorption capacities of the carbons while increasing the carbonization temperature led to a slight decrease in the amount of CO_2_ adsorption. The high carbonization temperature caused much more micropores to collapse and form larger micropores and mesopores. In addition, average pore size enlarged from 3.56 nm to 3.87 nm when temperature was increased from 766 °Cto 934 °C. It can be said that the most effective parameter on the surface areas and micropore volumes of the carbons synthesized in this study is the TEOS molar ratio. Therefore, the TEOS molar ratio was the most effective parameter, on the CO_2_ adsorption capacity of carbons, among the synthesis parameters examined.

The measured CO_2_ uptake values for the templated carbons synthesized in this study are close to the results presented in some previous studies in the literature while they are lower than others. Kim et al. synthesized three-dimensional hierarchical porous carbon materials by using furfuryl alcohol (PCM-F) or phloroglucinol (PCM-P) as carbon precursor and acid-labile mesoporous ZnO/Zn(OH)_2_ spheres as hard template. The CO_2_ uptakes of PCM-F and PCM-P are 1.83 and 2.55 mmol/g at 273 K, respectively [47]. Saha and Deng synthesized ordered mesoporous carbon from phloroglucinol by soft-template approach and measured adsorption properties of CO_2_, methane, nitrous oxide, and ammonia. The adsorption equilibrium capacity of the ordered mesoporous carbon synthesized of the CO_2_ is 1.49 mmol/g at 800 Torr and 298 K [48]. Tiwari et al. synthesized oxygen-enriched nanostructured carbon derived from resorcinol-formaldehyde by using mesoporous silica as template and reported the CO_2_ uptake of 1.5 mmol/g at 30 °C [27]. Shi et al. prepared hierarchically porous carbon frameworks with the combination of hard template and NaOH activation. For this carbon, the CO_2_ uptake at 273 K and 1 bar was reported as 3.80 mmol/g [49]. The nitrogen and sulfur dual-doped ordered mesoporous carbon spheres were prepared by Konnola and Anirudhan [50] and the capacity of 4.25 mmol/g at 273 and 1 bar was reported for CO_2_ uptake. In another research, the hierarchical porous carbons from sugar as the carbon precursor and nano-CaCO_3_ as the hard template were prepared and the results showed that the CO_2_ uptake reaches 2.84 mmol/g at 25 °C and 1 bar and 3.66 mmol/g at 0 °C and 1 bar [51]. According to these results, higher capacities were generally obtained for the activated or nitrogen, sulfur doped carbons. Besides the pore structure of carbons, surface chemistry is known to be an effective factor on the CO_2_ adsorption.

**Figure 10:**
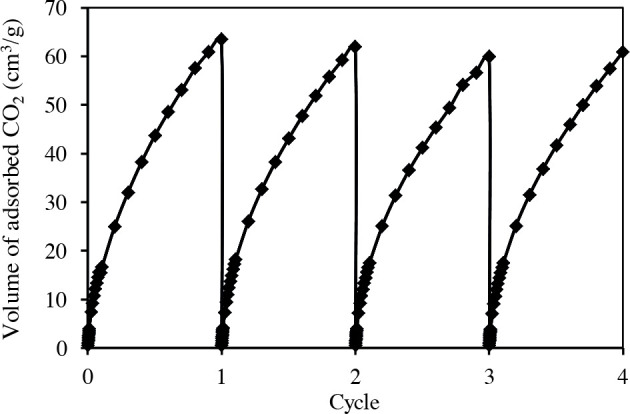
CO_2_ adsorption cycles obtained for the S-0.73/T-0.52/C-850 carbon.

The cyclic stability of the adsorbent was tested by repeated consecutive adsorption and desorption steps. The adsorption/desorption cycle of CO_2_ for S-0.73/T-0.52/C-850 carbon shows almost identical isotherm curves after four cycles (Figure 10). The average adsorption capacity was not changed during these cycles. These results indicate that the templated porous carbon is reversible and exhibits good adsorption stability. 

The organic-inorganic self-assembly can be achieved in the one-pot method. Thus, the silica template is formed simultaneously with the carbon. Since this process does not require silica hard template preparation prior to the carbon synthesis, it significantly simplifies the process compared to the traditional hard template process and saves time, labour and energy. At the same time, using a low-cost carbon precursor such as sucrose can make the process more economical. As a result, the carbons obtained in this study may be of interest as potential adsorbents in CO_2_ adsorption.

## 4. Conclusion

The templated porous carbons from sucrose were synthesized using one-pot method and characterized with various techniques such as Raman spectroscopy, FESEM, TEM, TGA, XRD, and N_2_ adsorption. The sucrose amount, TEOS molar ratio and carbonization temperature were changed during the synthesis of carbons and the influences of these parameters on the pore properties and CO_2_ adsorption of carbon materials were investigated. Results showed that the TEOS molar ratio is the most effective parameter on the pore properties of the synthesized carbons. The highest surface area of 1289 m^2^/g and the highest micropore volume of 0.467 cm^3^/g were measured for the carbon prepared with the lowest TEOS molar ratio. For this templated carbon, the highest CO_2_ uptake of 2.28 mmol/g was also measured. Hence, the templated porous carbons can be used as CO_2_ adsorbent. It is hoped that these results for CO_2_ uptake with the templated carbons provides the benefits for further researches on the related porous carbons for CO_2 _capture.
